# Unveiling the Mysteries of Contrast-Induced Acute Kidney Injury: New Horizons in Pathogenesis and Prevention

**DOI:** 10.3390/toxics12080620

**Published:** 2024-08-22

**Authors:** Zhong Wang, Qiuhan Wang, Xuezhong Gong

**Affiliations:** Department of Nephrology, Shanghai Municipal Hospital of Traditional Chinese Medicine, Shanghai University of Traditional Chinese Medicine, Shanghai 200071, China; 12023190@shutcm.edu.cn (Z.W.); m17730366659@163.com (Q.W.)

**Keywords:** contrast-induced acute kidney injury, contrast media, nephropathy, nephrotoxicity, review

## Abstract

The utilization of contrast media (CM) in clinical diagnostic imaging and interventional procedures has escalated, leading to a gradual increase in the incidence of contrast-induced acute kidney injury (CI-AKI). Presently, the scarcity of effective pharmacological treatments for CI-AKI poses significant challenges to clinical management. Firstly, we explore the pathogenesis of CI-AKI in this review. Beyond renal medullary ischemia and hypoxia, oxidative stress, cellular apoptosis, and inflammation, emerging mechanisms such as ferroptosis, release of neutrophil extracellular traps (NETs), and nitrosative stress, which offer promising avenues for the management of CI-AKI, are identified. Secondly, a comprehensive strategy for the early prevention of CI-AKI is introduced. Investigating the risk factors associated with CI-AKI is essential for the timely identification of high-risk groups. Additionally, exploring early sensitive biomarkers is crucial for early diagnosis. A synergistic approach that combines these sensitive biomarkers, CI-AKI risk factors, and disease risk prediction models enhances both the accuracy and efficiency of early diagnostic processes. Finally, we explore recent pharmacological and non-pharmacological interventions for the management of Cl-AKI. Beyond the traditional focus on the antioxidant N-acetylcysteine (NAC), we look at active compounds from traditional Chinese medicine, including tetramethylpyrazine (TMP), salvianolic acid B (Sal B), as well as emerging preventive medications like N-acetylcysteine amide (NACA), alprostadil, and others, which all showed potential benefits in animal and clinical studies for CI-AKI prevention. Furthermore, innovative strategies such as calorie restriction (CR), enhanced external counterpulsation (EECP), and mesenchymal stem cell therapy are highlighted as providing fresh insights into Cl-AKI prevention and management.

## 1. Introduction

Contrast-induced acute kidney injury (CI-AKI) is manifested by an abrupt decline in kidney function as a consequence of intravascular exposure to contrast media. It is also a common complication after coronary angiography (CAG) and percutaneous coronary intervention (PCI) [1]. Kidney Disease: Improving Global Outcomes (KDIGO) group defines CI-AKI as an increase in serum creatinine value to more than 1.5 times the baseline creatinine value within 7 days of contrast exposure; or an increase in serum creatinine of ≥26.4 μmol/L (0.3 mg/dL) within 48 h; or a urinary output of <0.5 mL/kg/h within 6 h of CM exposure [2]. With the advancement of medical imaging and the extensive utilization of interventional diagnostic and therapeutic techniques, the clinical application of CM has become increasingly prevalent, leading to a significant increase in the incidence of CI-AKI [3]. It is important to note that not all AKIs that occur after contrast administration are caused by the CM themselves. Many other risk factors for AKI should also be considered, including hypotension, hypovolemia, inflammation, and sepsis. AKI that occurs after CM use due to these factors is referred to as contrast-associated acute kidney injury (CA-AKI). Failure to differentiate between these risk factors in the clinical setting may result in delayed or inappropriate treatment. In healthy individuals, the incidence of CI-AKI is below 1%, indicating that kidneys without significant risk factors generally withstand contrast exposure well. However, in individuals with pre-existing renal insufficiency or additional risk factors, such as diabetes, hypertension, advanced age, and cardiovascular disease, the incidence of CI-AKI can escalate to 15% [4]. Given the potential for kidney damage associated with the use of CM, particularly in patients with pre-existing severe renal disease, physicians exercise caution in their application. Nonetheless, in the context of life-threatening emergencies such as ST-segment elevation myocardial infarction (STEMI), the utilization of CM is essential for conducting detailed diagnostic assessments and enabling timely endovascular interventions. While CI-AKI often manifests as transient renal injury, some individuals may suffer from irreversible renal decompensation, need hemodialysis, or, in severe cases, face mortality [5]. Studies have indicated that the in-hospital mortality rate for patients with CI-AKI is between 21 and 22%, while for patients without CI-AKI, the rate is significantly lower, ranging from 0.9 to 1.4% [6,7]. These findings suggest that CI-AKI may not only have potential long-term effects on renal function but also significantly increase in-hospital mortality rates. Currently, the absence of universally endorsed preventive and therapeutic strategies for CI-AKI, aside from extracellular volume expansion with NaCl or NaHCO_3_, underscores the significance of identifying and managing pertinent risk factors to mitigate the development of CI-AKI [8]. Drawing upon the research foundation of CI-AKI’s pathophysiology, this review presents early integrated prevention strategies focusing on risk factors, risk assessment models, and sensitive biomarkers. It also reviews recent preventive and therapeutic strategies for CI-AKI, along with exploring potential future medications and methods, aiming to offer a novel outlook on the treatment of CI-AKI.

## 2. Pathogenesis of CI-AKI

**Figure 1:**
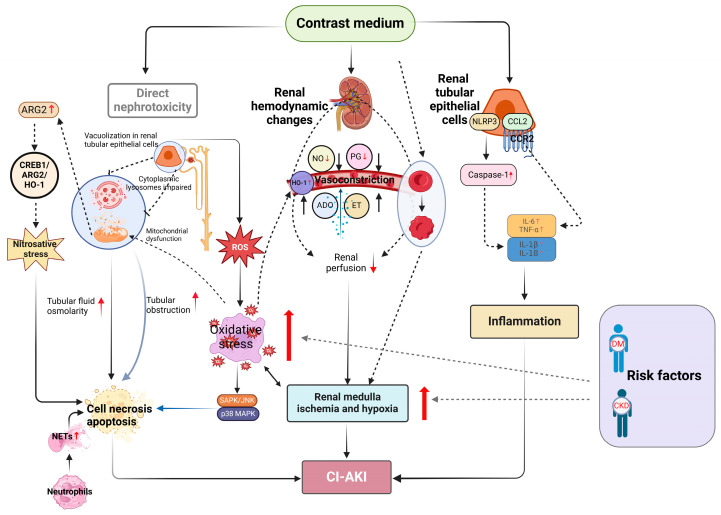
Schematic diagram of CI-AKI. Abbreviations: NO, Nitric oxide; PG, Prostaglandins; ET, Endothelin; ADO, Adenosine; HO-1, Heme oxygenase-1, ROS, Reactive oxygen species; DM, Diabetes mellitus; CKD, Chronic kidney disease.

The pathogenesis of CI-AKI is multifaceted, encompassing a range of cellular and molecular mechanisms that remain incompletely elucidated. Research indicates that the direct nephrotoxic effects of CM, renal medullary ischemia and hypoxia, oxidative stress, inflammation, and immune responses are intimately linked to the onset and advancement of CI-AKI. Additionally, the release of NETs, ferroptosis, and nitrosative stress are also implicated in the pathogenesis of CI-AKI. In the pathogenic cascade of CI-AKI, hypoxia may lie upstream of oxidative stress, nitrosative stress, NETosis, ferroptosis, and, to a large extent, even inflammation. In other words, hypoxia may be a key driver of these pathological processes. Notably, the critical role of predisposing risk factors in the development of CI-AKI cannot be overlooked when exploring its pathogenesis. These risk factors include, but are not limited to, pre-existing renal insufficiency, diabetes mellitus, and hypertension. These factors contribute to the onset and progression of CI-AKI in susceptible individuals by exacerbating mechanisms such as increased renal sensitivity to CM, decreased renal antioxidant capacity and repair mechanisms, and altered renal hemodynamics. Understanding these predisposing risk factors and their interactions with the pathogenesis of CI-AKI will enable a more comprehensive exploration of the disease’s complexity and provide a scientific basis for developing preventive and therapeutic strategies (Figure 1).

### 2.1. The Direct Nephrotoxicity of CM

Almost all CM have direct cytotoxic effects on vascular endothelial cells and renal tubular epithelial cells, characterized by swelling of the proximal renal tubular epithelial cells, vacuolar changes, and changes in cytoplasmic lysosomes, ultimately leading to cell necrosis [9]. Under an electron microscope, it can be observed that the polarity of the renal tubular epithelial cells is disordered and the mitochondrial membrane is damaged [10]. The cytotoxic effect is intensified under conditions of hypoxia, potentially due to plasma membrane damage and mitochondrial dysfunction triggered by ROS-mediated endoplasmic reticulum stress, alongside disturbances in intracellular calcium homeostasis [11]. Furthermore, the accumulation of CM creates a hypertonic environment conducive to apoptosis [12]. Upon CM administration, they remain within the vascular lumen, unattached to plasma proteins, facilitating their unhindered filtration through the glomerulus. CM are not reabsorbed in the tubules, leading to their progressive concentration as water and solutes are reabsorbed, significantly increasing their viscosity. This exposes renal tubular epithelial cells to elevated concentrations of CM for extended periods, exacerbating cellular damage and death. Reduced renal clearance of CM in patients with renal insufficiency may result in a greater accumulation of CM in the kidneys, potentially increasing the risk of CI-AKI [13]. As renal tubular epithelial cells perish and detach from the basement membrane, tubular occlusion ensues, prolonging the cytotoxic impact of CM and further compromising renal function [12].

### 2.2. Renal Medulla Ischemia and Hypoxia

On the one hand, after the CM enter the renal vessels, the dynamics of renal blood flow change; the renal vessels first undergo a brief and rapid dilation; the renal blood flow increases, and then the renal vessels strongly vasoconstrict, especially the vessels in the renal medullary region, leading to a decrease in the total renal blood flow [14,15]. Insufficient renal blood perfusion, a decrease in the glomerular filtration rate, and severe ischemia and hypoxia in the renal medulla lead to acute kidney injury. This contrast-induced hemodynamic change may be related to the synthesis and release of some vasoactive substances. Specifically, after intravascular injection of CM, plasma levels of atrial natriuretic peptide (ANP) increase rapidly, which may be associated with rapid, transient vasodilation. At the same time, the direct toxicity of CM to vascular endothelial cells results in an increase in endothelin and adenosine and a decrease in NO and prostaglandins, which may be associated with persistent vasoconstriction. When this balance shifts toward vasoconstriction, it results in renal medullary ischemia and hypoxia [14,16]. On the other hand, a transient early increase in the glomerular filtration rate (GFR) and urine flow following contrast administration, along with subsequent diuresis, increases solute delivery to the distal nephron and leads to increased oxygen consumption caused by enhanced tubular sodium reabsorption, particularly in the medullary thick limbs. This process also plays a central role in the development of medullary hypoxia and injury following contrast administration [14]. The third generation of CM, iso-osmolar CM, has high viscosity properties that increase the viscosity of urine and blood [17]. The increased concentration of CM in renal tubules increases urine viscosity, slowing urine flow and increasing intrarenal tubular pressure. This pressure can transfer to the renal interstitium, increasing interstitial pressure, compressing vasa recta, and consequently reducing blood flow to the renal medulla. Additionally, higher blood viscosity increases vascular resistance and slows blood flow, particularly in the renal medulla—a region with lower vascular density and inherently slower blood flow. Abnormal red blood cell shapes can further heighten blood viscosity, worsening renal medulla ischemia and hypoxia. In vitro experimental studies have revealed that CM can impact the cytoskeleton of erythrocytes, leading to alterations in the spectrin of the homogeneous reticular structure. This process results in the phenomenon of spectrin–actin co-localization, which induces the formation of echinocytes. Such changes contribute to microcirculatory disorders, exacerbating the hypoxia of renal tissues [18]. Notably, factors predisposing to CI-AKI such as diabetes or CKD are characterized by an already intensified medullary hypoxia [14]. And diabetes-induced microangiopathy and endothelial dysfunction may further reduce renal blood flow, thereby exacerbating renal medullary ischemia and hypoxia. These significantly increase the risk of CI-AKI in these patients [19].

### 2.3. Oxidative Stress and Apoptosis

Numerous studies have linked oxidative stress to the development of CI-AKI [20,21,22]. CM impact ATP synthesis by causing renal medulla ischemia and hypoxia, thereby disrupting medullary oxygen homeostasis. Specifically, hypoxic conditions reduce oxidative phosphorylation, leading to the production of large amounts of ROS. These ROS may directly damage cell membranes, proteins, and DNA, ultimately causing cell death or apoptosis. Notably, ROS-induced damage to mitochondrial DNA leads to mitochondrial dysfunction, which in turn increases ROS production, creating a positive feedback loop that intensifies oxidative stress. Renal tubular epithelial cells, with their high metabolic activity and mitochondria-rich composition, are particularly vulnerable to oxidative stress [23]. The accumulation of ROS in these cells might act as intracellular messengers, activating signaling pathways such as SAPK/JNK and p38 MAPK, which indirectly promote apoptosis [24,25]. Additionally, CM-induced elevated ROS levels may reduce the inhibition of Na/K-ATPase activity in the medullary thick limb, leading to its overactivation and further disrupting medullary oxygen homeostasis. This disruption of the Na/K-ATPase function not only affects the ionic balance of renal cells but also increases ATP consumption [26]. In the context of CI-AKI, this increased energy consumption may further damage renal cells already in a hypoxic state, exacerbating cellular dysfunction and death. Studies have indicated that elevated glucose concentrations may exacerbate CM-induced oxidative stress in mesangial cells. This observation suggests that diabetic patients could be at a higher risk of developing CI-AKI and underscores the significant role of predisposing risk factors in the pathogenesis of CI-AKI.

Apoptosis, a regulated process of cellular self-destruction, plays a crucial role in shielding the organism from various deleterious stresses. CM exposure may disrupt the equilibrium between pro-apoptotic and anti-apoptotic mechanisms, leading to renal function impairment. Yang et al. discovered that oxidative stress mediates CM-induced apoptosis in renal tubular epithelial cells through the mitochondrial pathway. This process is potentially linked to a deficiency in PINK1/Parkin-dependent mitochondrial autophagy, subsequently impacting cellular redox balance and energy metabolism. Such disruptions trigger mitochondrial dysfunction and membrane loss, resulting in altered mitochondrial permeability and the release of pro-apoptotic proteins, ultimately culminating in apoptotic cell death [27]. Moreover, ROS influence the activities of prostacyclin synthase and nitric oxide synthase, culminating in the decreased production of nitric oxide and prostaglandins. This reduction exacerbates renal medulla ischemia and hypoxia, creating a detrimental feedback loop. In conclusion, oxidative stress and apoptosis are intricately linked to the development of CI-AKI.

### 2.4. Inflammation and Immunity

Inflammation and immunity are crucial to the onset and progression of numerous acute and chronic kidney diseases [28]. It is broadly accepted that the genesis of CI-AKI is intimately associated with these factors, with inflammation alone potentially doubling the risk of CI-AKI [29]. A cross-sectional study indicated a significant association between inflammatory markers, including neutrophil-to-lymphocyte ratio (NLR), monocyte-to-lymphocyte ratio (MLR), neutrophil-to-lymphocyte*platelet ratio (NLPR), systemic inflammatory index (SII), and systemic inflammation response index (SIRI), and CI-AKI [30]. In experimental animal studies, researchers have documented that CM provoke immune responses, notably enhancing the migration of immune cells and the accumulation of cytokines. A marked elevation in inflammatory cytokines, specifically IL-6 and TNF-α, along with renal dysfunction and tubular damage, has been observed in experimental animals following in vivo administration of CM [31]. Our prior research also identified elevated levels of CCL2 and CCR2, alongside significant increases in IL-6 and TNF-α in the kidneys of rats within a CI-AKI model exposed to CM. Pre-treatment with TMP and NAC mitigated their upregulation, further underscoring the pivotal role of inflammation in the pathogenesis of CI-AKI [10]. The NLRP3 inflammasome, acting as a pattern recognition receptor, is pivotal in activating immune responses by detecting pathogens and damage signals. Upon perceiving alterations in the intracellular milieu, such as increased ROS, NLRP3 initiates the assembly of inflammatory vesicles to incite an immune response. Activation of caspase-1 by NLRP3, which in turn cleaves and activates pro-inflammatory cytokines like interleukin-1β and interleukin-18, induces an inflammatory response that culminates in cell death [32,33]. A study demonstrated that administering exogenous klotho, a protein known for its multifunctional ability to counter oxidative stress and inflammation, ameliorated renal function in CI-AKI mice. This improvement was attributed to the inhibition of oxidative stress, inflammation, and the NF-κB/NLRP3-mediated inflammatory pathway, along with the downregulation of NLRP3, caspase-1, among others, suggesting a significant contribution of intrarenal inflammatory/immune responses to the pathogenesis of CI-AKI [34].

### 2.5. Ferroptosis

Ferroptosis, an iron-dependent programmed cell death characterized by fatal lipid peroxidation due to lipid ROS accumulation, plays an increasingly recognized role in AKI development. Intervention strategies blocking ferroptosis have demonstrated efficacy in mitigating renal ischemia/reperfusion injury. Recently, our team observed the occurrence of ferroptosis in renal tubular epithelial cells in both in vivo and in vitro models of CI-AKI, characterized by the accumulation of Fe^2+^, lipid peroxidation, and decreased activity of glutathione peroxidase 4 (GPX4). By employing Fer-1 and DFO, two classic inhibitors of ferroptosis, we found that they effectively enhanced cell viability and significantly reduced the production of ROS. Furthermore, we discovered that pre-treatment with TMP significantly inhibited renal dysfunction, prevented the production of ROS, reduced the accumulation of Fe^2+^, and promoted the expression of GPX4. These findings provide an important experimental basis for a deeper understanding of the molecular mechanisms of CI-AKI and the development of new therapeutic strategies [35].

### 2.6. Neutrophil Extracellular Traps

NETs, comprising DNA and antimicrobial substances, are released by neutrophils in response to pathogens or inflammation. The release process, known as NETosis, confines pathogen spread and aids in their elimination. Although the early initiation of NETosis supports swift pathogen removal, an imbalance in this protective mechanism may endanger the organismal homeostasis. Research indicates that neutrophils, upon infiltration, can be induced to form NETs in models of ischemic kidney injury, exacerbating renal damage [36]. Recent findings reveal NETs’ role in CI-AKI’s development and progression by affecting endothelial cells within the renal microcirculation. The expression level of NETs correlates positively with CI-AKI severity, and inhibiting NETs diminishes renal cell apoptosis and pyroptosis, and mitigates inflammation [37]. Furthermore, following CM administration, neutrophils are promptly activated and recruited, potentially marking NETs as early biomarkers for CI-AKI diagnosis.

### 2.7. Nitrosative Stress

Nitrosative stress, characterized by an overaccumulation of nitrite within intra- and extracellular environments, parallels oxidative stress in its capacity to inflict cellular damage. Affecting cell fate through various mechanisms, excessive nitrosative stress directly initiates apoptosis and may further amplify it indirectly by causing mitochondrial damage and disrupting signaling pathways, contributing to diverse disease pathogeneses [38]. In recent research, ARG2 expression significantly increased in a CI-AKI mouse model, predominantly accumulating in the mitochondria of renal tubular cells. ARG2 elevation during CI-AKI fosters nitrosative stress, precipitating renal tubular cell apoptosis. Specifically, ARG2-mediated nitrosative stress aggravates CI-AKI by activating the CREB1/ARG2/HO-1 signaling pathway [39], suggesting nitrosative stress as a critical pathogenic mechanism in CI-AKI.

## 3. Strategies for the Prevention and Treatment of CI-AKI

Currently, there is no specific treatment available for CI-AKI, underscoring the importance of prevention as the primary strategy for managing CI-AKI. The paramount strategy for preventing CI-AKI involves the early identification of individuals at high risk, managing pertinent risk factors, assessing morbidity risk, and minimizing unnecessary diagnostic imaging. The pursuit of sensitive biomarkers is crucial for the early diagnosis of CI-AKI. For patients requiring CM, morbidity risk can be mitigated through judicious CM use and personalized hydration strategies. Furthermore, pharmacological prevention can be achieved with the administration of antioxidants, renoprotective agents such as statins, hemodynamic modulators, along with emerging drugs aimed at reducing oxidative stress, diminishing inflammation, and curtailing the apoptotic response [40]. Non-pharmacological measures, including remote ischemic preconditioning, hemodialysis, and other innovative preventive strategies, also play a vital role in CI-AKI prevention (Figure in Section 3.3.6) (Table in Section 3.3.6).

### 3.1. An Integrated Strategy for Early Prevention of CI-AKI

#### 3.1.1. Identifying High-Risk Groups

**Figure 2:**
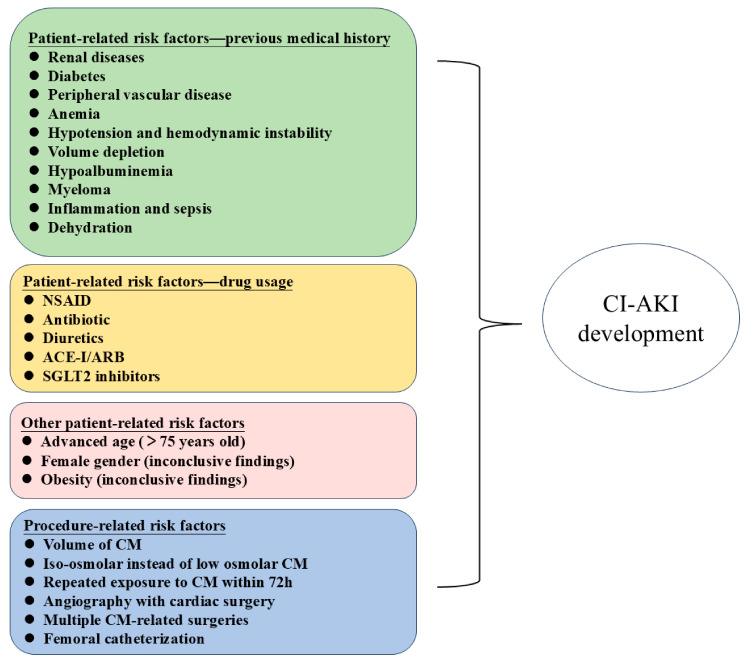
The main risk factors for CI-AKI.

In mitigating CI-AKI, the precise recognition of associated risk factors is crucial for decreasing its incidence and formulating efficacious prevention measures. Pre-existing severe renal insufficiency emerges as the paramount risk factor for CI-AKI. Studies have indicated that the incidence of CI-AKI is over threefold higher in patients with chronic kidney disease (eGFR < 30 mL/min/1.73 m^2^) compared to those with normal renal function [41], a disparity possibly attributed to diminished adaptive capacity or heightened cellular susceptibility. Consequently, for patients at high risk, an angiography should be circumvented when feasible, opting instead for alternative diagnostic modalities such as intravascular ultrasound to reduce contrast-induced nephrotoxicity [42]. Furthermore, in patients with renal insufficiency who require the use of CM, a preference for CM with low toxicity is advised, ensuring that the administered dose is kept within the minimal effective threshold. As research advances, an increasing number of risk factors for CI-AKI have been identified, necessitating targeted preventive measures against these risks (Figure 2).

#### 3.1.2. Finding Sensitive Biomarkers

Additionally, the timely diagnosis of CI-AKI is pivotal for patient outcomes. Serum creatinine is a commonly used diagnostic marker for CI-AKI, but its delayed increase and interference from non-renal factors lead to an inability to accurately and promptly reflect kidney damage [43]. Consequently, the identification of novel biomarkers for the early diagnosis of CI-AKI is essential. We have compiled findings on several potential biomarkers [44], predominantly associated with the pre-injury stage, injurious, and other relevant biomarkers, as illustrated in Table 1. Although the development of such biomarkers appears promising, their clinical integration for CI-AKI diagnosis has been hampered by the absence of standardized thresholds and a lack of clinical validation across large cohorts [45].

#### 3.1.3. Risk Stratification through Predictive Modeling

Based on risk factors and sensitive biomarkers, researchers have constructed multiple predictive models to evaluate the incidence risk of CI-AKI. The majority of these studies have employed discriminative and calibrated techniques to ascertain the internal validity of the models, thereby offering a dependable framework for the risk assessment and prevention of CI-AKI. Ideal predictive models ought to feature parameters that are readily obtainable, possess substantial predictive accuracy, and be applicable preoperatively. Various risk models, differing in their levels of precision and complexity, have been established, as depicted in Table 2. While these models are capable of forecasting the incidence of CI-AKI, their clinical utility is limited, necessitating additional validation. It is important to note that these risk-scoring models were primarily designed for patients undergoing endovascular interventions, where relatively large amounts of CM are used. In contrast, contrast-enhanced CT (CECT) requires only a small amount of CM. According to propensity score-matched studies, the risk of CI-AKI after CECT is generally low in most patients but remains significant in those with advanced CKD [68]. Therefore, the applicability and accuracy of existing scoring systems require further evaluation for this specific patient population. Additionally, research indicates that a combination of multiple measures, including the Mehran score, L-FABP, and NGAL, significantly enhances the specificity of CI-AKI predictions [69], underscoring that a synergistic diagnostic approach yields greater accuracy, thereby enabling early diagnosis and therapeutic intervention.

### 3.2. Pharmacological Strategies

#### 3.2.1. Antioxidant

**Figure 3:**
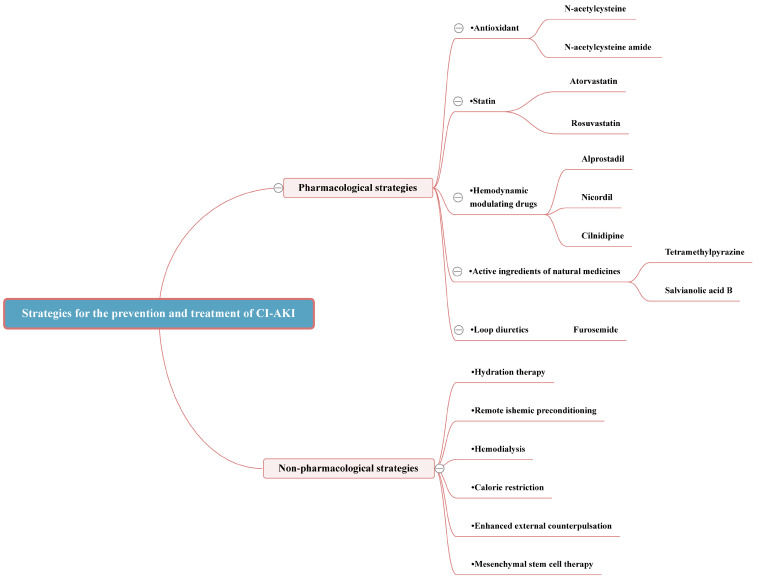
Strategies for the prevention and treatment of CI-AKI.

N-acetylcysteine (NAC), a precursor to the antioxidant glutathione (GSH), plays a pivotal role in cellular defense against oxidative stress through its unique sulfhydryl-containing structure. NAC primarily promotes its antioxidant effects by facilitating GSH synthesis, stabilizing nitric oxide levels, and influencing the microcirculation within the renal cortex and medulla, thereby mitigating contrast-induced renal vasoconstriction [75]. NAC is widely employed in the prevention and treatment of CI-AKI due to its low cost, wide availability, ease of administration, and lack of significant adverse effects (Figure 3). Recent meta-analyses of randomized clinical trials (RCTs) have demonstrated that NAC usage moderately lowers the incidence of CI-AKI and enhances renal function [76] (Table 3). However, clinical practice has shown that NAC has low bioavailability in vivo, which may be related to its limited intracellular and mitochondrial penetration capacities. Therefore, the efficacy of NAC in CI-AKI prevention remains a subject of ongoing research, with its actual effectiveness and optimal application yet to be conclusively determined.

N-acetylcysteine amide (NACA), an amide derivative of NAC, demonstrates enhanced lipophilicity, membrane permeability, and antioxidant capabilities [77]. Recent investigations have revealed significant antioxidant actions of NACA in AKI and nervous system diseases [78,79,80]. Our prior research has also established that NACA surpasses NAC in efficacy for preventing CI-AKI, both in vivo and in vitro. This augmented effect appears to be mediated by the modulation of oxidative stress and the regulation of thioredoxin-1 (Trx1), which in turn inhibits the apoptosis signal-regulating kinase 1 (ASK1) and the p38 mitogen-activated protein kinase (MAPK) pathway as well as by preventing apoptosis in renal tubular cells [81]. Consequently, NACA may serve as a superior renal protective agent against CI-AKI compared to NAC.

Other emerging antioxidants, such as febuxostat, coenzyme Q10, trimetazidine, and edaravone, show promise as clinical candidates for CI-AKI prevention, warranting further investigation [82,83,84].

#### 3.2.2. Statin

Prior research has revealed that statins possess protective properties that may prevent CI-AKI [85]. These benefits likely stem from antioxidative actions and the suppression of inflammation, pyroptosis, and apoptosis, among other mechanisms. An animal study has shown that atorvastatin can inhibit the TLR4/MyD88/NF-κB signaling pathway, help improve renal tubular epithelial cell function, and reduce injury, inflammation, and burn death [86]. Additionally, a meta-analysis of five randomized controlled trials (RCTs) assessing the impact of rosuvastatin on preventing CI-AKI in patients with acute coronary syndrome revealed a notable benefit. Specifically, preoperative rosuvastatin therapy significantly reduced the risk of CI-AKI in patients at high risk for chronic kidney disease or diabetes [87]. Similarly, another meta-analysis investigating the effects of atorvastatin on preventing CI-AKI showed that high-dose atorvastatin preconditioning was significantly associated with a reduced prevalence of CI-AKI in patients undergoing CAG [88].

#### 3.2.3. Hemodynamic Modulating Drugs

Alprostadil, a synthetic analogue of prostaglandin E1 (PGE1), is recognized for its vasodilatory effects, which effectively dilate renal blood vessels and inhibit both platelet aggregation and thrombosis. Additionally, alprostadil reduces proteinuria and enhances kidney function by boosting blood flow to the kidneys and increasing the glomerular filtration rate [89]. One clinical study demonstrated that alprostadil in combination with conventional therapy was effective in preventing CI-AKI in patients with renal dysfunction undergoing PCI [90]. Moreover, another study indicated that alprostadil could serve as a prophylactic therapy in patients at high risk of developing complications according to Mehran’s risk score, likely owing to its anti-inflammatory effects [91].

Nicordil, is the first ATP-sensitive potassium channel opening agent for clinical use. It promotes dilation of the systemic vasculature, particularly the arterioles, thus enhancing organ function through improved perfusion, which includes significant benefits to the kidneys. An RCT demonstrated that oral administration of nicorandil could reduce the incidence of CI-AKI without increasing the incidence of postoperative adverse events. In patients with renal dysfunction at high risk, preoperative oral nicorandil administration may also prevent CI-AKI occurrence [92].

Cilnidipine, a calcium channel blocker, has shown renoprotective effects, significantly enhancing renal function and alleviating apoptosis, oxidative stress, and mitochondrial damage in renal tubular cells caused by iohexol, both in vitro and in vivo [93]. Yet, the current research relies on traditional experimental approaches and singular dosage applications, differing from the long-term medication needs in clinical practice. The actual efficacy, optimal dosage, and treatment timing of cilnidipine in CI-AKI prevention and treatment remain to be elucidated through future prospective clinical trials.

However, evidence from some studies suggests that vasodilators such as dopamine, nifedipine, fenoldepam, and endothelin receptor antagonists have not demonstrated preventive effects on CI-AKI [44,94,95], indicating the need for further research and validation in the use of vasodilators for CI-AKI prevention and treatment.

#### 3.2.4. Active Ingredients of Natural Medicines

Given the current stagnation in Western medicine research concerning the prevention and pathogenesis of CI-AKI, exploring traditional Chinese medicine (TCM) and Chinese herbal medicine presents a promising alternative. Several active compounds derived from these traditional sources have demonstrated potential in both preventing and treating CI-AKI.

TMP, an alkaloid derived from *Ligusticum sinense* ‘*Chuanxiong*’, is renowned for its protective role against kidney injuries caused by gentamicin, diabetes mellitus, and ischemia–reperfusion injury. Our team’s prior research has shown that TMP counters CM-induced renal injury by inhibiting p38 MAPK activation. Further investigations revealed TMP’s capacity to reverse CM-induced activation of the CCL2/CCR2 pathway, ameliorate renal oxidative stress and abnormal mitochondrial dynamics, and regulate mitophagy in tubular cells, underscoring TMP’s renoprotective effects [10,96].

Sal B, a water-soluble compound from *Salvia miltiorrhiza*, has demonstrated protective effects against CI-AKI in several studies. These investigations have highlighted its ability to mitigate oxidative stress, endoplasmic reticulum stress (ERS), and inflammation in renal cells [97,98,99]. Sal B’s effectiveness is attributed to its activation of the Nrf2 pathway, reduction in oxidative stress markers, and inhibition of apoptosis-related proteins. Sal B modulates key signaling pathways, including PI3K/Akt/Nrf2 and TLR4/NF-κB/NLRP3, which play significant roles in its renoprotective actions. In addition, a clinical study has also highlighted the potential benefit of Sal B in preventing CI-AKI after PCI. The results indicated that patients treated with Sal B experienced a lower incidence of CI-AKI and displayed better protection of renal function when compared with controls hydrated with normal saline (NS) alone [100]. These findings suggest that Sal B can reduce the harmful effects of CM and could potentially offer a new treatment for preventing CI-AKI.

In summary, active ingredients of natural medicines exhibit potential in preventing CI-AKI, offering new avenues for its management. However, the clinical application of many such treatments remains limited, with several still undergoing experimental evaluation or lacking robust evidence for widespread use. Future research could leverage modern pharmacological technologies to further explore traditional Chinese medicine, blending the strengths of both to develop more effective and widely applicable compounds.

### 3.3. Non-Pharmacological Strategies

#### 3.3.1. Hydration Therapy

Hydration therapy is a cornerstone in the prevention of CI-AKI, aiming to dilute the nephrotoxic effects of CM and maintain renal perfusion. Despite its widespread acceptance, debates persist regarding the optimal hydration strategy, including the type of fluid (sodium chloride vs. sodium bicarbonate), the timing (pre-procedural vs. post-procedural hydration), and the rate of administration. Some studies advocate for the use of sodium bicarbonate over sodium chloride based on its potential to reduce oxidative stress within the renal tubules. However, subsequent research, including randomized controlled trials and meta-analyses, has produced mixed results [85]. While pre-procedural hydration is generally recommended to ensure optimal renal perfusion before contrast exposure, the optimal duration and rate of hydration to maximize renal protection without causing fluid overload, especially in patients with heart failure or compromised renal function, are not definitively established. Moreover, the role of oral hydration as a supplementary or alternative approach to intravenous hydration has been explored, with some studies suggesting its potential effectiveness and convenience. However, the comparative efficacy of oral versus intravenous hydration in CI-AKI prevention is still under investigation [101,102]. Given these controversies, individualized risk assessment and tailored hydration protocols based on patient-specific factors such as baseline renal function, volume status, and presence of comorbid conditions are advocated by many experts. The RenalGuard System, a device used to prevent CI-AKI, helps prevent hypovolemia and minimize potential renal damage from CM by accurately controlling the infusion rate and maintaining a high urinary flow rate during contrast examinations. This is particularly beneficial for individuals at high risk of CI-AKI. One possible mechanism by which furosemide-matched hydration therapy with the RenalGuard System may reduce the risk of CI-AKI is that it blocks tubular sodium reabsorption in the medulla, thereby reducing oxygen consumption by the cells, and enhances contrast dilution in the renal tubules by increasing urine flow [103,104]. Further research is still needed to refine hydration therapy and reduce the associated controversy.

#### 3.3.2. Remote Ischemic Preconditioning

Remote ischemic preconditioning (RIPC) is an innovative and noninvasive approach, the essence of which lies in augmenting the resilience of vital organs against prolonged ischemic assaults by inducing transient ischemia–reperfusion in a location remote from these central organs. It has been documented that transient muscle ischemia can confer protection, notably to cardiac and renal tissues [105]. The protective mechanism of RIPC is believed to involve diminishing inflammatory responses, oxidative stress, and apoptosis [106,107]. Meta-analyses have demonstrated that the incidence of CI-AKI in patients undergoing RIPC prior to surgery was significantly reduced compared to those who did not undergo RIPC (6.5% vs. 13.5%, *p* < 0.001) [108]. However, certain studies have indicated that RIPC does not safeguard diabetic patients from CI-AKI, hinting that the effectiveness of RIPC in CI-AKI prevention may vary depending on the patients’ underlying conditions [109]. Notably, RIPC has yet to be associated with any adverse events in clinical trials, positioning it as a safe, novel, noninvasive, and cost-effective preventive strategy potentially beneficial in lowering the incidence of AKI among high-risk patients.

#### 3.3.3. Hemodialysis

Hemodialysis, a prevalent treatment for renal failure, is notably effective in removing CM from blood circulation, with its efficacy well established [110,111]. However, the suitability of hemodialysis for preventing CI-AKI remains contentious. While initial studies have shown some promising outcomes [112,113], preventive hemodialysis has not gained broad acceptance, possibly due to inappropriate dialysis timing or inadequate ultrafiltration. Importantly, for patients undergoing chronic hemodialysis or peritoneal dialysis, additional dialysis post-contrast media administration is not advised. This caution stems from the potential of the dialysis procedure to trigger inflammatory responses and release vasoactive substances, thereby elevating the risk of CI-AKI. Furthermore, the KDIGO guidelines advise against employing hemodialysis or hemofiltration solely for CM removal as a preventative measure against CI-AKI. Thus, the application of hemodialysis in CI-AKI prevention warrants meticulous consideration.

#### 3.3.4. Calorie Restriction

Calorie restriction (CR) is a well-documented strategy for extending the lifespan across various animal species, with CR-activated nutritional signals playing a critical role in injury prevention [114]. CR enhances aging kidneys’ adaptability to hypoxia and ameliorates age-related structural and functional kidney damage [115]. The CR treatment group exhibited reduced apoptosis, oxidative stress, and inflammation levels compared to the normal caloric intake CM group. CR’s mechanism involves mitigating CI-AKI in renal tissue through the SIRT1/GPX4 pathway [4], offering a side-effect-free, non-genetic, and environmental intervention beneficial for patients at high CI-AKI risk.

#### 3.3.5. Enhanced External Counterpulsation

Enhanced external counterpulsation (EECP), a novel noninvasive therapy, demonstrates significant benefits in treating coronary artery disease and angina pectoris. By applying continuous external pressure waves, EECP improves cardiac blood supply, alleviates heart disease symptoms, and enhances life quality. EECP has been shown to boost renal perfusion, mitigate renal injury [116], and enhance vascular endothelial function by increasing plasma nitric oxide levels and decreasing plasma endothelin-1 concentrations [117]. These effects, improving microcirculatory disturbances, present valuable prospects for CI-AKI prevention and treatment. Furthermore, EECP induces atrial natriuretic peptide secretion, enhancing diuresis, reducing tubular fluid viscosity, and minimizing CM exposure duration [118], underscoring its multifaceted benefits in cardiovascular and renal protection. A recent clinical study demonstrated that EECP treatment effectively reduces serum creatinine levels, increases eGFR, and lowers the incidence of CI-AKI in patients with CKD and diabetes mellitus undergoing CAG or PCI [119].

#### 3.3.6. Mesenchymal Stem Cell Therapy

Mesenchymal stem cells (MSCs), multipotent adult stem cells sourced from adipose tissue, bone marrow, umbilical cord blood, and placenta, exhibit notable anti-inflammatory, anti-fibrotic, and anti-apoptotic properties [120]. Recent studies underscore that MSCs cultured in serum-free conditions (SF-MSCs) have significantly higher anti-inflammatory and anti-fibrotic capabilities. In disease models, MSC application has positively impacted various conditions, notably improving renal function and reducing renal tubular cell apoptosis rates in CI-AKI models, potentially through the suppression of cleaved caspase 3 expression. Additionally, in vitro research suggests SF-MSCs mitigate apoptosis via paracrine effects. Nonetheless, experiments involving the downregulation of epithelial growth factor (EGF) through small interfering RNA (siRNA) have diminished the renoprotective and anti-apoptotic impact of SF-MSCs in CI-AKI models [121], highlighting SF-MSCs’ potential in CI-AKI prevention and treatment and suggesting new therapeutic directions.

**Table 3:** Evidence of pharmacological and non-pharmacological strategies for the prevention and treatment of CI-AKI in experimental and clinical settings.

Type of Strategy	Intervention	Study Type	Study Design	Study Results	Ref.
Pharmacological strategies	NAC	Meta-analysis	Multiple RCTs	The risk of CI-AKI and Scr levels are significantly decreased with NAC use.	[76]
	NACA	Experimental	In Vivo Animal Model	NACA may protect against CI-AKI through modulating Trx1 and ASK1/p38 MAPK pathway to result in the inhibition of apoptosis among renal cells.	[81]
	Trimetazidine	Clinical	RCT	Trimetazidine along with isotonic saline infusion is more effective than isotonic saline alone in reducing the risk of CI-AKI in patients with pre-existing renal dysfunction.	[122]
	Edaravone	Experimental	In Vivo Animal Model	Edaravone may prevent and alleviate renal impairment and oxidative stress in rat models of CI-AKI by improving renal antioxidant capacity.	[83]
	Febuxostat	Clinical	RCT	Febuxostat has a renoprotective effect, and it can help to reduce the incidence of CI-AKI in CKD stage 3 patients undergoing PCI.	[84]
	CoQ10	Experimental	In Vivo Animal Model	CoQ10 presented an antioxidant effect on the CI-AKI in male diabetic rats by improving renal function and renal hemodynamics, preserving morphology and reducing oxidative stress.	[123]
	Atorvastatin	Experimental	In Vivo Animal Model	Atorvastatin can inhibit the TLR4/MyD88/NF-κB signaling pathway, help improve renal tubular epithelial cell function, and reduce injury, inflammation, and burn death.	[86]
	Rosuvastatin	Meta-analysis	Multiple RCTs	Preoperative rosuvastatin therapy significantly reduced the risk of CI-AKI in patients at high risk for chronic kidney disease or diabetes.	[87]
	Alprostadil	Clinical	RCT	For renal insufficiency patients undergoing PCI, the associative usage of alpromazil with routine treatment can effectively prevent CI-AKI.	[90]
	Nicordil	Clinical	RCT	Oral administration of nicorandil could reduce the incidence of CI-AKI without increasing the incidence of postoperative adverse events.	[92]
	Cilnidipine	Experimental	In Vivo Animal Model	Cilnidipine markedly improved kidney function and alleviated tubular cell apoptosis, oxidative stress, and mitochondrial damage induced by iohexol in vitro and in vivo.	[93]
	TMP	Experimental	In Vivo Animal Model	TMP’s capacity to reverse CM-induced activation of the CCL2/CCR2 pathway ameliorate renal oxidative stress and abnormal mitochondrial dynamics and regulate mitophagy in tubular cells.	[10]
	Sal B	Clinical	RCT	Sal B reduces the incidence of CI-AKI and protects renal function after PPCI, and the effects were superior to those of NS hydration.	[100]
Non-pharmacological strategies	Hydration therapy	Meta-analysis	Multiple RCTs	Furosemide with matched hydration by the RenalGuard System may reduce the incidence of CI-AKI in high-risk patients undergoing PCI or TAVR.	[104]
	RIPC	Meta-analysis	Multiple RCTs	The incidence of CI-AKI in patients undergoing RIPC prior to surgery was significantly reduced compared to those who did not undergo RIPC.	[108]
	Hemodialysis	Clinical	RCT	For patients with chronic renal failure who are undergoing PCI, periprocedural hemofiltration given in an ICU setting appears to be effective in preventing the deterioration of renal function due to CI-AKI.	[113]
	CR	Experimental	In Vivo Animal Model	CR protected CI-AKI via SIRT1/GPX4 activation. CR may be used to mitigate CI-AKI.	[124]
	EECP	Clinical	RCT	EECP increases the contrast clearance and may have an effect in reducing the risk of CI-AKI.	[119]
	MSC therapy	Experimental	In Vivo Animal Model and In Vitro Cellular Model	SF-MSCs might improve CI-AKI by exerting anti-apoptotic effects in a paracrine manner.	[121]

Abbreviations: NAC, N-acetylcysteine; NACA, N-acetylcysteine amide; CoQ10, Coenzyme Q10; TMP, Tetramethylpyrazine; Sal B, Salvianolic acid B; TAVR, Transcatheter aortic valve replacement; RIPC, Remote ischemic preconditioning; CR, Calorie restriction; EECP, Enhanced external counterpulsation; MSCs, Mesenchymal stem cells.

## 4. Conclusions and Prospects

The incidence of CI-AKI has risen alongside advancements in imaging medicine and interventional diagnostic and treatment technologies. Currently, beyond NaCl or NaHCO_3_ hydration, there is no globally recognized protocol for its prevention. To effectively combat CI-AKI, further comprehensive research into its pathogenesis and preventive measures is imperative. Recent years have seen significant strides in CI-AKI research, uncovering new mechanisms such as the release of neutrophil extracellular traps, ferroptosis, and nitrosative stress. Investigations into potential novel pharmacological treatments like TMP, Sal B, and NACA, alongside innovative approaches such as calorie restriction, enhanced external counterpulsation, and mesenchymal stem cell therapy, have opened new avenues for CI-AKI management. Nevertheless, several challenges remain in CI-AKI prevention and management.

Discrepancies in CI-AKI definitions and diagnostic criteria across various studies and guidelines complicate consensus on treatment. In clinical practice, the lack of specific biomarkers and the close temporal proximity of contrast agent use to other factors (surgery, other medications, etc.) complicate the differentiation between CI-AKI and CA-AKI. Many studies have misclassified CA-AKI as CI-AKI, leading to an inaccurate assessment of the true incidence and severity of CI-AKI. This misclassification may result in inappropriate therapeutic strategies, resource wastage, and a potentially negative impact on patient outcomes. Therefore, future research should focus on developing more accurate diagnostic tools and biomarkers to enhance the differentiation between CI-AKI and CA-AKI. In addition, in clinical practice, it has been observed that in some cases, renal function returns to baseline levels after a transient decline, and the occurrence of renal function recovery may lead to an underestimation of the true incidence of CI-AKI [125]. Additionally, when assessing CI-AKI, the impact of the renal functional reserve (RFR) should not be overlooked. The RFR refers to the kidneys’ ability to adapt and compensate for injury. Patients with a reduced RFR may be more susceptible to developing CI-AKI after contrast exposure [126]. However, increased creatinine levels may not necessarily indicate structural renal injury from CI-AKI but could instead reflect a reduction in RFR, making the kidneys more sensitive to injury or stress. For high-risk groups, such as those with CKD, relying solely on biomarkers is insufficient; dynamic observation and increased frequency of testing are effective means of improving CI-AKI detection. By closely monitoring changes in renal function, we can detect CI-AKI earlier and implement timely interventions.

While many studies endorse hydration therapy, debates continue over optimal fluid management practices and their patient-specific applicability, underscoring the necessity for personalized treatment approaches. In patients with heart failure, the reduced pumping capacity of the heart leads to decreased renal perfusion, resulting in water and sodium retention and subsequent kidney injury [127]. These high-risk patients are particularly susceptible to CI-AKI after exposure to CM. In this scenario, relying solely on rehydration therapy to prevent CI-AKI could potentially exacerbate the workload on the heart and increase the filtration demand on the kidneys, thereby posing additional risks. To address this, the combined use of furosemide is a strategy worth exploring. Although furosemide can be potentially nephrotoxic, it may offer nephroprotective benefits in specific populations at high risk for CI-AKI. First, it enhances contrast dilution and excretion in the renal tubule through increased urine flow, which reduce intraluminal transit time and renal tissue uptake. Second, it blocks tubular sodium reabsorption in the medulla, which reduces cellular oxygen consumption and thus protects the kidneys from hypoxic insult following radiocontrast [128]. In fact, furosemide alone (without hydration) was found to prevent medullary thick limb hypoxic injury in a CI-AKI animal model [129]. Finally, it also helps alleviate heart failure by reducing water and sodium retention through its diuretic effect [130]. Meanwhile, there are also studies suggesting that furosemide may increase the risk of CI-AKI [131,132], potentially due to inadequate rehydration and dehydration leading to volume depletion, which diminishes its pharmacologic effects. After conducting an in-depth review and analysis of the studies mentioned above, we have to note that furosemide-induced high-volume diuresis, combined with matched rehydration to maintain intravascular volume, could be a viable strategy to prevent and treat CI-AKI in specific high-risk populations, such as patients with heart failure or those undergoing PCI or TAVR.

Additionally, the decision to opt for alternative imaging techniques, particularly in high-risk patients, remains contentious. There are a number of non-iodinated CM currently available. Gd chelates, primarily used in magnetic resonance imaging (MRI), offer advantages such as low plasma protein binding and rapid clearance, resulting in a lower incidence of CI-AKI in patients with CKD. However, in patients with severely impaired renal function, Gd chelates carry the risk of nephrogenic systemic fibrosis (NSF) and should be used cautiously [133]. Carbon dioxide, a non-iodinated contrast medium, poses no allergic risk and is non-toxic to the kidneys, making it a common choice for angiography. A study by Tim Jakobi et al. demonstrated that carbon dioxide is a suitable and safe alternative to iodinated contrast media, particularly for intravascular remodeling in patients with peripheral arterial disease and CKD [134]. However, its use is limited to site-specific vascular imaging, such as lower limb vessels, and it is not suitable for imaging the heart and brain. Additionally, nanoparticles (e.g., cerium oxide nanoparticles and iron oxide magnetic nanoparticles) offer excellent imaging performance with reduced renal toxicity due to their nanoscale structure and special surface modifications [135]. However, certain types may trigger immune responses or adverse reactions upon accumulation, and their widespread use requires further research and clinical validation.

Finally, drug prevention efficacy remains a focal area of research; although some medications have demonstrated potential in animal models, their transition to clinical trials or robust clinical validation remains lacking. As we confront CI-AKI, delving into its specific mechanisms to devise more precise preventive measures and risk assessments, as well as exploring novel therapeutic interventions to mitigate or repair contrast-induced renal damage, remains crucial. We anticipate further breakthroughs in CI-AKI prevention and treatment through persistent efforts.

## Figures and Tables

**Table 1:** Potential biomarkers of CI-AKI.

Category	Biomarker	Molecular Weight (kDa)	Function	Significant Change in CI-AKI	Cutoff for CI-AKI Prediction	Ref.
Pre-injury phase biomarkers	DKK3(U)	30	Promote renal tubulointerstitial fibrosis through modulation of the canonical Wnt/b-catenin signaling pathway	NA	DKK3/creatinine ratio > 1.7 pg/mg	[46]
	β2M(S)	11.8	Indicator of renal tubular injury	NA	>2.8 mg/L at baseline	[47]
	HSP27(S)	22	Protect cells against different forms of cellular stress, including oxidative stress, as well as apoptosis	NA	<19.67 μg/L	[48]
	GGT(S)	68	Responsible for the extracellular catabolism of glutathione, a major component of intracellular antioxidant protective mechanisms	NA	>26.5 U/L	[49]
	Antithrombin III(S)	58	ATIII shows anti-inflammatory properties and also enhances renal blood flow.	NA	ATⅢ activity < 75%	[50]
Damage biomarkers	Cys-C(S)	13	Indicator of reduced kidney function	8 h	rise ≥ 10% at 24 h	[51]
	MK(S)	13	Regulates cell growth, cell survival, migration and anti-apoptotic activities in nephrogenesis and development.	2 h	NA	[52]
	IL-18(U)	18	Indicator of renal tubular injury	6 h	815.61 pg/mL at 12 h	[53]
	KIM-1(U)	85	A potential biomarker of proximal tubular injury	6 h	>366 ng/mL	[54]
	NGAL(U)	25	A potential biomarker of distal tubular injury	6 h	>20 ng/mL	[55]
	NAG(U)	>130	Indicator of renal tubular injury	24 h	NA	[56]
	L-FABP(U)	14	L-FABP can detect renal hemodynamic change following administration of CM.	24 h	≥24.5 μg/g Cr	[57]
Other biomarkers	MHR(S)	NA	Correlated with inflammation and has a prognostic value in patients with renal diseases.	NA	>0.95 × 10^9^/mmol	[58]
	DIN(S)	NA	A novel biomarker for severity of systemic and local inflammatory states	NA	>1.8% on ED admission	[59]
	GDF-15(S)	40	In response to oxidative stress, endothelial dysfunction, inflammation, and tissue injury, GDF-15 expression was increased.	NA	NA	[60]
	hs-CRP(S)	22.5	hsCRP is associated with an increased risk of CI-AKI.	NA	hs-CRP > 5 mg/DL	[61]
	SII(S)	NA	A relatively novel inflammatory marker combining platelet, neutrophil, and lymphocyte counts	NA	SII > 1282	[62]
	C-Peptide(S)	NA	C-Peptide has a renoprotective effect in diabetic nephropathy.	NA	≤2.39 ng/mL	[63]
	WBV(S)	NA	WBV is related to shear stress, atherosclerosis, and adverse cardiac events. WBV may affect renal function.	NA	WBV < 14.90	[64]
	TyG index(S)	NA	A reliable and specific biomarker for insulin resistance and is associated with renal dysfunction	NA	TyG index > 9.043	[65]
	CAR(S)	NA	An acute-phase reactant and is known to be associated with poor outcomes in predicting the development of CI-AKI	NA	NA	[66]
	AIP(S)	NA	Demonstrates plasma atherogenicity by combining TG and HDL-C in a single logarithmic fraction	NA	API > 0.62	[67]

Abbreviations: DKK3, Urinary dickkopf-3; β2M, β2Microglobulin; HSP27, Heat shock protein 27; GGT, Gamma-glutamyl transferase; Cys-C, Cystatin C; MK, Midkine; IL-18, Interleukin-18; KIM-1, Kidney injury molecule-1; NGAL, Neutrophil gelatinase-associated lipocalin; NAG, N-Acetyl-β-D-glucosaminidase; L-FABP, Liver-type fatty acid-binding protein; MHR, Monocyte to high-density lipoprotein ratio; DNI, Delta neutrophil index; GDF-15, Growth differentiation factor-15; hs-CRP, Pre-procedural high-sensitivity C-reactive protein; SII, Systemic immune-inflammation index; WBV, Whole blood viscosity; TyG, Triglyceride-glucose; CAR, C-reactive protein/albumin ratio; AIP, Atherogenic index of plasma.

**Table 2:** Risk stratification models.

Population Characteristics	Development Dataset	Variables Included in Models	No. of Risk Factors	Risk Stratification	C Statistic	Reference
PCI at one hospital	5571	Hypotension	8	Low (≤4) Moderate (5–8) High (9–12) Very-high (≥13)	0.67	[70]
		IABP				
		Heart failure				
		CKD				
		Diabetes				
		Age > 75 years				
		Anemia				
		Contrast volume				
PCI at one hospital	1500	Age ≥ 70	9	Low (≤7) Moderate (8–12) High (13–16) Very-high (≥17)	0.82	[71]
		Prior MI				
		Diabetes				
		Hypotension				
		LVEF ≤ 45%				
		Anemia				
		eGFR ≤ 45 (mL/min/1.73 m^2^)				
		HDL < 1 mmol/L				
		Urgent PCI				
CAG, PCI, or CECT at one hospital	7040	Baseline eGFR	13	NA	0.91	[72]
		RDW				
		Triglycerides				
		The most recent SCr Before the procedure				
		HDL				
		Total cholesterol				
		LDL				
		BU				
		P-LCR				
		Serum sodium				
		PCT				
		INR				
		BG				
CECT at one hospital	2240	DM	4	Low (0–2) Intermediate (3–4) High (5–6)	0.73	[73]
		Serum albumin level < 4.3 mg/dL				
		CKD stage 5				
		CKD stage 4				
PCI at three hospitals	931	Hematocrit	15	Low (<10) Moderate (10–16) High (>16)	0.84	[67]
		WBC				
		Platelet count				
		MCV				
		MCHC				
		RDW				
		MPV				
		Sodium				
		Potassium				
		Bicarbonate				
		Calcium				
		Glucose				
		Creatinine				
		Age				
		Gender				
PCI at one hospital	23,703	Age	7	NA	0.8	[74]
		CKD				
		Hematocrit				
		Troponin I				
		Blood urea nitrogen				
		Base excess				
		NT-proBNP				

Abbreviations: IABP, intra-aortic balloon pump; CKD, chronic kidney disease; MI, myocardial infarction; LVEF, left ventricular ejection fraction; HDL, high-density lipoprotein cholesterol; LDL, low-density lipoprotein cholesterol; BSA, body surface area; TIA, transient ischemic attack; WBC, white blood cell; RDW, red cell distribution width; BU, blood urea; P-LCR, platelet larger cell ratio; PCT, plateletocrit; INR, international normalized ratio; BG, blood glucose; MCV, mean corpuscular volume; MCHC, mean corpuscular hemoglobin concentration; MPV, mean platelet volume; NT-proBNP, N-terminal pro-brain natriuretic peptide.

## Data Availability

Data are contained within the article.
